# High concentration and yield production of mannose from açaí (*Euterpe oleracea* Mart.) seeds via mannanase-catalyzed hydrolysis

**DOI:** 10.1038/s41598-019-47401-3

**Published:** 2019-07-29

**Authors:** Alvaro Ferreira Monteiro, Ingrid Santos Miguez, João Pedro R. Barros Silva, Ayla Sant’Ana da Silva

**Affiliations:** 1 Biocatalysis Laboratory, National Institute of Technology, Ministry of Science, Technology, Innovation and Communication, Rio de Janeiro, 20081-312 RJ Brazil; 2 Federal University of Rio de Janeiro, Department of Biochemistry, Rio de Janeiro, 21941-909 RJ Brazil

**Keywords:** Biocatalysis, Hydrolases

## Abstract

The açaí seed corresponds to approximately 85% of the fruit’s weight and represents ~1.1 million metric tons of residue yearly accumulated in the Amazon region, resulting in an acute environmental and urban problem. To extract the highest value from this residue, this study aimed to evaluate its chemical composition to determine the appropriate applications and to develop conversion methods. First, mannan was confirmed as the major component of mature seeds, corresponding to 80% of the seed’s total carbohydrates and about 50% of its dry weight. To convert this high mannan content into mannose, a sequential process of dilute-acid and enzymatic hydrolysis was evaluated. Among different dilute-H_2_SO_4_ hydrolysis conditions, 3%-acid for 60-min at 121 °C resulted in a 30% mannan hydrolysis yield and 41.7 g/L of mannose. Because ~70% mannan remained in the seed, a mannanase-catalyzed hydrolysis was sequentially performed with 2–20% seed concentration, reaching 146.3 g/L of mannose and a 96.8% yield with 20% solids. As far as we know, this is the highest reported concentration of mannose produced from a residue. Thus, this work provides fundamental data for achieving high concentrations and yields of mannose from açaí seeds, which could add commercial value to the seeds and improve the whole açaí productive chain.

## Introduction

The *Euterpe oleracea* Mart. palm plant—otherwise known as the açaí palm—is a widely distributed plant in northern South America, predominantly found in the delta region of the Amazon river^1^. *E. oleracea* Mart. fruits—the açaí— are globose, measuring about 1.5 cm in diameter, green when immature, and dark purple when ripe^2^. In the past 15 years, the commercialization of açaí pulp has experienced an economic boom, with a sizable increase seen in the Brazilian and international markets, such as the United States, Japan, and Europe^3,4^. Brazil is the main açaí producer in the world, with more than 1,274,000 metric tons of açaí processed in 2017 (http://sidra.ibge.gov.br and http://www.sedap.pa.gov.br). The commercialized product—the açaí pulp—represents approximately only 15% of the mature fruit’s weight, whereas the açaí seed accounts for the other 85%^5,6^.

Consequently, the rapid increase of açaí commercialization has generated an enormous amount of açaí seeds as a by-product. Açaí can be produced during the whole year, however studies related to microregions in the State of Pará (the main açaí producer State) concluded that the highest production period occurs in the second half of the year^7^. Considering the current annual production of açaí and the proportion of the seed in relation to the whole fruit, it is estimated that more than 1,000,000 metric tons of seeds are deposited yearly in the Amazon region, with the prospect of more in the coming years. Today, only a small amount of the seeds is utilized for animal feed, plantations, or home gardens and crafts, and very few appropriate disposal methods currently exist, resulting in an acute environmental and urban problem^3^. It is of great environmental and economic interest to avoid waste production and simultaneously find new applications for açaí seeds, thus adding value to the productive chain and promoting local and social development. In this scenario, different studies have shown that açaí seeds can be a source of extracts rich in polyphenols with potential biological activities^8–10^ and its use for electric power generation has also been assessed^11^.

To extract the highest possible value from this residue and determine the appropriate applications, it is of the utmost importance to know its composition, but few systematic studies have been carried out with açaí seeds. Previous works have reported that açaí seeds contain high amounts of carbohydrates (~70%), with cellulose being the main polysaccharide^12–16^. In contrast, Rambo *et al*.^17^ have shown that 53.8% of the seed is composed of mannan, a polymer of mannose. This high mannan content renders this seed as a valuable and unexplored material. Therefore, the study of processing methods to release mannose from açaí seeds and the confirmation of its actual composition are extremely relevant.

Up to now, there are no reports of studies aiming to release mannose from açaí seeds, which is a sugar with a high potential to be a functional ingredient that exhibits biological functions of great interest in the cosmetic, pharmaceutical, and food industries^18–20^. For example, mannose can be easily reduced to mannitol—a specialty chemical with a wide variety of uses in the pharmaceutical industry—in a process with a 90% yield^21^.

One alternative to explore the açaí seed’s potential is to develop mild methods that efficiently release sugars from the material. Sugars derived from hemicellulose can be directly released from plant cell walls by applying different methods, such as acid, thermal, enzymatic hydrolysis, or microbial fermentation^22^. Commonly, dilute inorganic acids, including sulfuric and hydrochloride acid, have been employed to hydrolyze the xylan-containing hemicelluloses of some lignocellulosic biomasses with more than 90% efficiency, resulting in a liquid fraction rich in free xylose. Nevertheless, some studies have indicated that mannan-containing hemicelluloses may be less prone to the action of dilute sulfuric acid in a single step hydrolysis^23^. To overcome the low yields of mannan hydrolysis by diluted acids, mannan-degrading enzymes could be applied as a strategy to increase the release of free mannose from mannan-rich residues^24–26^, which can also be combined in a sequential step after a mild dilute-acid hydrolysis. The enzymatic hydrolysis of mannan main-chain involves β-mannanase and β-mannosidase as degrading enzymes. β-Mannanases, or 1,4-β-D-mannan mannohydrolase, are endo-type enzymes which catalyze the random hydrolysis of internal β-1,4-D-mannopyranosyl linkage, initiating the degradation of mannan polysaccharides to release short β-1,4-mannooligomers with 2~10 monosaccharide units and to produce new chain ends. β-Mannosidases, on the other hand, are exo-type enzymes that cleave β-1,4-linked mannosides, releasing mannose from mannans and mannooligosaccharides^27–29^. The enzymatic process could permit to work with mild conditions that are less damaging to the environment, besides improving the yield using simple protocols^30^.

Considering that açaí seeds can be (i) a potential rich source of mannan, (ii) its high abundance in Brazil, (iii) the environmental impact of its accumulation in Amazon region, and (iv) the limited knowledge about this waste product, the aim of the current study was to explore the potential of açaí seeds as feedstock by evaluating its carbohydrate composition and developing acidic- and enzymatic-catalyzed strategies to maximize mannose production, thus aiming to give a proper destination to the seeds while adding value to the whole açaí productive chain.

## Results and Discussion

### Açaí seed chemical characterization

**Figure 1:**
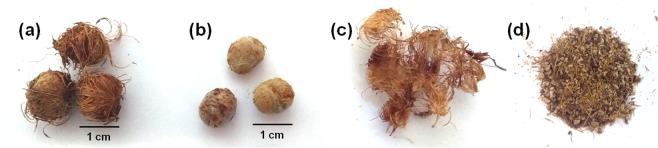
Açaí seed samples: (**a**) whole seeds; (**b**) core stone after removing the external fibers; (**c**) fiber layer; (**d**) milled whole seeds.

Two lots of açaí seeds were characterized in the current study. Samples from lot 1 were received as shown in Fig. 1a and were noted as “whole seeds” (Fig. 1a), while lot 2 contained samples already milled (Fig. 1d). For characterization, the whole seeds samples from lot 1 were processed with a knife mill as received. First, 35 samples of dried mature açaí seeds were weighted to determine the proportion of the mass of the external fiber layer (Fig. 1c) to the whole seed samples (Fig. 1a). By botanical definition, the external fiber layer is not considered part of the seed; however, we denominated the seed as the residue generated after the depulping and sieving of açaí (fibers + seed) because—for the sake of brevity—it is improbable that any large-scale commercial use of this residue will separate those fractions.

The average weight of the whole seeds was 0.78 g ± 0.12, ranging from 0.56 g to 1.06 g, and the mass percentage of fiber in relation to the whole seed was equivalent to 5.97% ± 1.45. These data are in close agreement with a previous study that reported that the whole seeds average weight was 0.72 ± 0.04 g and that the fibrous layer corresponded to 6.50% of the whole seed weight^5^.

The literature data regarding the açaí seed composition so far is conflicting. Therefore, to better evaluate the seed’s uses, a confirmation of its chemical composition is crucial to design the most suitable processing methods for sugar recovery. In the current study, the characterization of two distinct seed samples lots was performed, as well as an analysis of different seed fractions. Table 1 presents the composition of the milled samples of whole seed from the two lots and of the core stones and fiber layer of one lot. The composition of the whole seed showed that the material is mostly composed of carbohydrates, with mannan—a polymer of mannose—being its main component and corresponding to 47.09% and 52.46% of its total dry weight for lot 1 and lot 2, respectively. Smaller amounts of other structural sugars were also identified, such as glucose, xylose, galactose, and arabinose. Rambo *et al*.^17^ reported a similar composition for açaí seeds, corresponding to 53.6% mannose, 8.66% glucose, 3.18% xylose, 1.43% galactose, 0.69% arabinose, and 0.17% rhamnose. The seed’s lipid content was not analyzed in the current study; however, it has been reported that *Euterpe oleracea* Mart. seeds contain only 0.33% total fat^16^.

**Table 1:** Chemical composition of the whole açaí seeds from two different lots and of the core stone and fiber layer of one lot, here expressed as a percentage of dry matter.

Component	Dry mass (%)			
	whole seed (lot 1)	Core stone (lot 1)	Fiber layer (lot 1)	whole seed^a^ (lot 2)
Anhydromannose	47.09 ± 1.42	47.19 ± 2.58	nd^c^	52.46 ± 1.51
Anhydroglucose	6.09 ± 0.67	4.61 ± 0.48	21.88 ± 0.46	8.40 ± 0.52
Anhydroxylose	1.83 ± 0.33	1.13 ± 0.16	15.12 ± 0.39	2.05 ± 0.22
Anhydrogalactose	1.79 ± 0.21	2.61 ± 0.12	0.90 ± 0.04	1.51 ± 0.27
Anhydroarabinose	0.40 ± 0.02	0.85 ± 0.03	0.82 ± 0.03	0.63 ± 0.03
AIS^b^	18.34 ± 0.64	18.36 ± 0.61	31.80 ± 0.36	19.54 ± 1.56
Extractives	15.45 ± 0.95	16.72 ± 2.43	12.89 ± 1.88	9.89 ± 2.09
Ash	0.61 ± 0.09	0.41 ± 0.03	2.12 ± 0.06	0.44 ± 0.02

^a^The characterization of the core stones and fibers are shown only for lot 1 because lot 2 was received milled from the producer. ^b^AIS: acid insoluble solids account for the organic matter that was insoluble after acid hydrolysis condition and is calculated by not counting the amount of acid insoluble ash. ^c^nd: not detected.

The composition of the core stones (Fig. 1b) showed a high similarity with the whole seed, as expected, considering that this fraction corresponds to almost 94% of the whole seed’s mass. The fibers, however, presented a distinct sugar profile with no detectable mannose content and higher contents of glucan, xylan, and acid insoluble solids (AIS) when compared with the core stone. AIS can be presumably counted as lignin; however, because açaí seeds are quite different from typical lignocellulosic biomass, further analyses of these AIS are required to confirm if all of its content corresponds to the lignin. The high percentage of extractives in açaí seeds is in accordance with its reported polyphenolic polymeric procyanidins content^31^. Nevertheless, it is possible that not all the content of the polymeric procyanidins is accounted for in the extractives because hydrogen bonds can be formed between the hydroxyl groups of polyphenols and oxygen of the glycosidic linkages of polysaccharides, making procyanidins imprisoned in the cell wall of carbohydrates and not extractable using organic solvents^32^. Further investigations would be needed to elucidate if this occurs in açaí seeds.

The high mannan content confirmed in the current study contradicts most studies reporting on açaí seeds’ composition, which have stated cellulose as the main polysaccharide in the seed^12–16^. Although Rambo *et al*.^17^ quantified the carbohydrate content of açaí seeds and reported mannan as the main polysaccharide, no further discussion was made about this finding in that study. The fact that many studies reported high cellulose content in the seed could be related to the use of indirect methods to determine the material’s composition, which measure the total fiber content instead of specific sugar quantification using chromatographic methods. For example, Altman^12^ employed a method developed by Waksman and Stevens^33^ which is based on the principle that, in general, hemicellulose is hydrolyzed upon treatment with dilute acid, while cellulose and lignin are resistant. However, because the mannan from the açaí seeds is—much like cellulose—highly recalcitrant toward dilute-acid hydrolysis, mannan has been incorrectly quantified as cellulose. Wycoff *et al*.^16^ have analyzed açaí seed samples by nuclear magnetic resonance and observed peaks related to glycosidic bonds, inferring that these were related to cellulose and hemicellulose and citing previous studies that indicate cellulose as the seed’s main polysaccharide.

**Figure 2:**
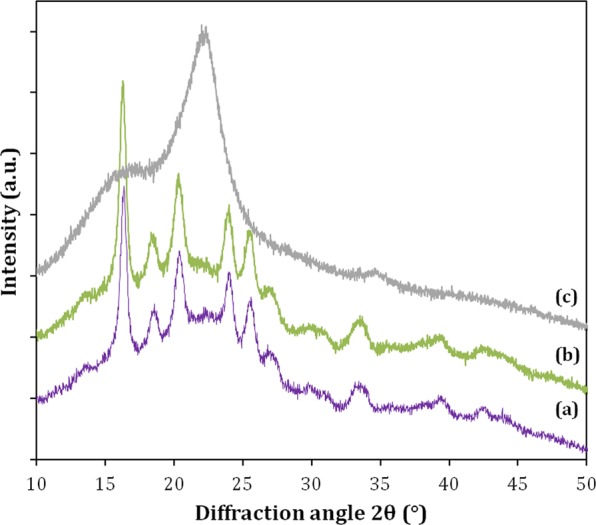
XRD profiles of the milled samples of (**a**) whole açaí seed; (**b**) açaí seed core stone, and (**c**) açaí seed fibers.

Cellulose has an unusual crystallinity among biopolymers, and X-ray diffraction (XRD) is the most commonly used technique to obtain data for cellulose crystallinity. Therefore, XRD analyses were performed to verify if the açaí seed samples had the typical diffraction profile of materials containing high amounts of cellulose. Figure 2 presents the XRD profiles of the milled samples of the whole açaí seeds (stone plus fibers), the açaí seed stone, and the fibers.

The açaí seed fiber presented a typical cellulose I diffraction pattern with two peaks at 2*θ* equal to 16.0 and 22.0, which correspond to the cellulose crystal planes 110 and 200, respectively^34^. The fibers’ XRD profile was very similar to the ones found for other agricultural residues, such as sugarcane bagasse and wheat straw, which are biomasses that contain ~40% cellulose^35^. These data are in accordance with the macroscopic aspect of the fibrous material, as well as with its high glucan content. However, the samples of milled whole seeds or açaí stones did not show a diffraction peak corresponding to the cellulose crystalline plane 200. A comparison of the diffraction patterns, which were completely different in the characteristic region of cellulose crystals (15°–25°), also corroborates the sugar composition data for each fraction analyzed, confirming that açaí seeds do not contain cellulose as their main polysaccharide.

This high mannose content in açaí seeds is supported by the fact that the secondary walls of the endosperm cells in the seeds of many species contain very little cellulose^36^; these consist of noncellulosic cell wall storage polysaccharides that are digested during germination, being usually mannans, galactomannans, glucomannans, xyloglucans, and galactans^28,37,38^. A number of botanical studies have identified mannan as a customary reserve polysaccharide in seeds from the Arecaceae (palm trees) family^39–41^. However, these studies have focused on the aspects of plant physiology and seed germination and have not quantitatively evaluated the seed’s polysaccharide chemical composition. Even though mannans can be common cell wall storage polysaccharides, it is interesting that the açaí seed’s mannan content of about 50% of its dry weight is quite high. Most vegetal biomasses, such as grasses and hardwoods, have low amounts of mannose (<2%), while softwood can reach up to 15%^22^. Palm kernel cakes, copra meal, and spent coffee grounds are agro-industrial residues with the highest reported mannose content, reaching 30–35%, 28–32%, and 14–19% of their dry weight, respectively^24,42–46^.

The reported chemical structure and composition of other mannose-rich seeds or residues indicate that the most common polysaccharides in these materials are β-1,4-mannan and/or galactomannan, which has a (1 → 4)-β-D-mannopyranosyl backbone and can alternatively be substituted by α-D-galactopyranosyl residues at position *O*-6^28,36^. For example, the endosperm of many seeds from small leguminous trees, as well as the locust bean and guar gums, have galactomannan as the main polysaccharide, with a galactose:mannose molar ratio of 1:2 to 1:4^28^. In contrast, the mature palm kernel, coconut copra meal, ivory nuts, and green coffee beans have mannans composed of linear chains of 1,4-linked β-D-mannopyranosyl residues that contain less than 5% galactose and small amounts of other polysaccharides^37^. Therefore, considering the monosaccharide’s profile obtained from the acid hydrolysis of açaí seeds (Table 1) and the data from the literature for other seeds, it is hypothesized that a linear β-1,4-mannan is the main polysaccharide of the mature açaí seed. The very low content of galactose in mature açaí seeds renders the presence of galactomannan unlikely because this polysaccharide is reported to have a galactose:mannose molar ratio of 1:2. Additionally, it is known that seeds with deposits of linear mannan in their endosperm are very resistant to mechanical damage due to its high insolubility in water^47^. The evaluation of the açaí seed fracture resistance resulted in an average minimal force required to rupture the seeds of 966 N ± 237 (98.5 kgf) (Supplementary Table 1), which is higher than the value found for hard-seeded genotypes of pomegranate^48^. Therefore, this high resistance of açaí seeds to compression also supports the presence of linear mannan in their endosperm. However, further studies to elucidate the carbohydrate structure will be necessary.

### Effect of acid hydrolysis for mannose release from açaí seeds

The processing strategies to release mannose from mannan will depend on the polysaccharide structure. The commercial gums, like locust bean, guar and tara gums, contain galactomannan as their major component. To extract these galactomannans, the endosperm is separated from the seed hulls and germ, being subsequently milled and washed with ethanol and hot water to separate the galactomannan^49^. Linear mannans, however, are usually insoluble in water. Mannan from the ivory nut, for example, is classified in I and II, Mannan I can be extracted with alkali and Mannan II is insoluble in aqueous NaOH^28^. In the current study, moderate dilute-acid hydrolysis was evaluated as a possible strategy to release mannose from açaí seeds through the breakdown of the mannan, making the monomeric sugars readily available in the acid’s liquid phase. Acid hydrolysis was evaluated at a fixed temperature of 121 °C and by varying the H_2_SO_4_ concentration and residence time. Table 2 presents the severity factor for each condition evaluated, the percentage of insoluble solids recovered, and its chemical composition and the sugar composition of the hydrolysates obtained.

**Table 2:** Characterization of the recovered insoluble solids and sugar composition of the hydrolysates from the acid hydrolysis of açaí seeds at different H_2_SO_4_ concentrations and residence times.

% H_2_SO_4_ (m/m)	Time (min)	% RIS^a^	R_0_^b^	Content in recovered solid (%)				Sugar concentration in the hydrolysate (g/L)				
				Mannan	Glucan	Xylan	AIS^c^	Man	Gal	Xyl	Ara	Glu
*Un* ^*d*^	—	100	—	52.46 ± 1.51	8.40 ± 0.52	2.05 ± 0.22	19.54 ± 1.56	—	—	—	—	—
1.5%	30	81.1 ± 0.1	0.99	60.39 ± 1.67	10.01 ± 0.10	2.46 ± 0.20	23.72 ± 0.94	13.54 ± 2.10	2.12 ± 0.22	1.03 ± 0.16	1.58 ± 0.03	0.51 ± 0.01
3.0%	30	73.5 ± 0.4	1.19	57.32 ± 0.72	8.99 ± 1.21	1.38 ± 0.19	22.39 ± 0.88	23.28 ± 1.65	2.71 ± 0.12	1.88 ± 0.07	1.38 ± 0.02	0.67 ± 0.03
3.5%	30	71.6 ± 0.1	1.32	55.80 ± 1.32	9.72 ± 0.15	0.83 ± 0.09	27.84 ± 1.38	27.65 ± 0.37	3.01 ± 0.08	2.21 ± 0.01	1.41 ± 0.06	0.78 ± 0.08
4.5%	30	71.6 ± 0.7	1.29	53.36 ± 1.12	10.13 ± 0.21	1.05 ± 0.04	29.22 ± 1.54	29.93 ± 1.83	2.98 ± 0.18	2.48 ± 0.33	1.31 ± 0.08	0.84 ± 0.02
1.5%	60	72.8 ± 0.5	1.30	58.99 ± 0.99	9.66 ± 0.05	1.49 ± 0.10	27.28 ± 1.43	27.12 ± 0.13	3.14 ± 0.03	2.57 ± 0.13	1.68 ± 0.02	0.87 ± 0.01
3.0%	60	70.2 ± 0.2	1.44	54.10 ± 2.20	10.28 ± 0.76	0.98 ± 0.10	27.07 ± 0.57	41.76 ± 1.09	3.55 ± 0.05	3.45 ± 0.24	1.50 ± 0.03	1.07 ± 0.06
3.5%	60	70.4 ± 0.6	1.64	51.34 ± 3.10	10.32 ± 0.56	0.81 ± 0.14	25.05 ± 2.28	46.22 ± 0.44	3.58 ± 0.06	3.26 ± 0.21	1.48 ± 0.03	1.14 ± 0.11
4.5%	60	62.3 ± 0.9	1.70	55.25 ± 1.18	10.42 ± 0.40	0.40 ± 0.07	30.86 ± 3.09	49.54 ± 0.20	3.38 ± 0.02	3.10 ± 0.34	1.39 ± 0.02	1.34 ± 0.12

^a^RIS: recovered insoluble solids; ^b^R_0_: combined severity factor; ^c^AIS: acid insoluble solids; ^d^Un: untreated açaí seed from lot 2.

From Table 2, there is a correlation between the severity of the acid hydrolysis, the percentage of insoluble solids recovered, and the concentration of mannose released, indicating that to a certain extent, more biomass components are transferred into the liquid phase when the severity is higher, which was expected. The lowest severity condition (R_0_ 0.99) resulted in 81.1% recovered solids, while for the most severe condition, this value decreased to 62.3%. The duration of the treatment, from 30–60 min, had an important impact on the release of sugars. Mannose concentration increased according to the increase in acid concentration from 1.5% to 4.5% H_2_SO_4_, ranging from 13.54 g/L to 29.93 g/L (9.4% to 20.7% yield) for hydrolysis carried out for 30 min and from 27.12 g/L to 49.54 g/L (18.8% to 34.4% yield) when treatments were carried out for 60 min. The same pattern could be observed for glucose. Similarly, the dilute-acid hydrolysis of nondilapidated spent coffee grounds—a residue rich in mannan—resulted in 85–70% solids recovery after hydrolysis, with acid concentrations ranging from 1–5% v/v and residence times from 30–60 min at 95 °C^50^.

**Figure 3:**
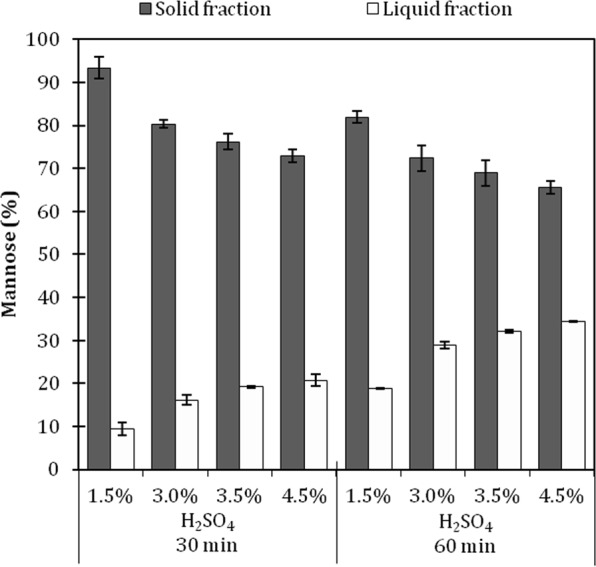
Percentage of mannose recovery from the milled açaí seeds after acid hydrolysis with dilute H_2_SO_4_ (1.5%–4.5%) for 30 and 60 min of retention time at 121 °C. White bars: Percentage of mannose recovered in the liquid fraction after acid hydrolysis. Dark gray bars: mannose content retained in the solid fraction after acid hydrolysis.

Even though mannose and glucose concentrations increased with a higher severity, the xylose, arabinose and galactose concentrations in the hydrolysates decreased during the most severe conditions, indicating the partial degradation of these sugars in the liquid fraction. This is in accordance with studies that reported a lower activation energy for the hydrolysis of xylan (101 kJ/mol) than for mannan (113 kJ/mol) and cellobiose (110 kJ/mol) hydrolysis^51–53^. It is well known that the combination of high temperature, acidic pH, and prolonged reaction time may contribute to the formation of undesired compounds derived from sugar dehydration, such as furfural and hydroxymethylfurfural, as well as the degradation of phenolic structures^54,55^. Therefore, to evaluate acid hydrolysis conditions, one should take into account both the sugar release yield and formation of degradation products. Moreover, it is also important to calculate the total mannose recovery because higher severity treatments may result in a higher release of mannose, but also in a partial degradation of this sugar, causing an overall loss of the desired product. In the present study, low amounts of hydroxymethyl furfural were quantified in the hydrolysates and were equivalent to 56 mg/L, which was detected only in the most severe condition (4.5% H_2_SO_4_, 60 min). Very low concentrations of acetic acid were quantified in the hydrolysates from all conditions, which were in the range of 60–210 mg/L. Other compounds, such as furfural, vanillin, gallic, ferulic, cinnamic, and hydrobenzoic acids were monitored but were either under the limit of quantification or not detected. Figure 3 shows the mannose recovery balance in both the solid and liquid fractions obtained after the acid hydrolysis of seeds for each condition. Biomass characterization protocols are multistep and very laborious procedures, but overall, over 95% of the original mannose content could be detected either in the hydrolysate or preserved in the solid fraction, which is in good agreement with the absence/low concentration of the degradation products detected (Fig. 3). The high mannose recovery indicates that at the temperature evaluated, the acid concentration and reaction duration were at an acceptable range for mannose stability.

Optimized dilute-acid hydrolysis of xylans at mild temperatures can reach yields of 80–95%, while cellulose is much more resistant to dilute acid attacks at temperatures under 200 °C^56^. Açaí seed mannan’s susceptibility to dilute-acid hydrolysis seems to lie in between because 34.3% of the mannan could be converted into mannose during the most severe condition evaluated. Reducing sugars when in solution exist as a mix of cyclic structures, which are more resistant to degradation, and as open chains that are more reactive acyclic carbonyl forms^57^. In polysaccharides, the sugar at the chain end or ramified end will exist in ring and open chain structures, being more susceptible to degradation^53^. Additionally, it has been shown that, at room temperature, glucose and mannose present a lower carbonyl percentage than galactose, xylose, and arabinose^58^. These data correlate to the low concentration of the sugar degradation products found in the hydrolysates and to the fact that water-insoluble and linear mannans, which are likely cellulose, can form crystalline structures^59,60^ that are recalcitrant and resistant to dilute sulfuric acid attack; however, other amorphous hemicelluloses, such as galactoarabinoxylans, are known to be highly susceptible to dilute-acid hydrolysis^56^.

The current knowledge of the chemical hydrolysis of mannan is limited because there are only a few reports of mannose production from vegetal biomass through acid hydrolysis. One study^44^ evaluated the dilute sulfuric acid hydrolysis under microwave irradiation of deproteinated palm kernel cake (PKC) containing 55.71% mannan; the results of that study showed a mannose yield of 92% at the optimized condition of 148 °C, 0.75 N H_2_SO_4_ (equivalent to 3.5% w/w), 10.5 min, and a solid:liquid (S:L) ratio of 1:50. Besides the use of microwave irradiation assistance, higher temperatures and the possible differences between deproteinated PKC and açaí seed recalcitrance to acid attack, the higher mannose yields achieved for PKC acid hydrolysis may have been favored by the low substrate concentration evaluated (S:L ratio of 1:50). In the present study, a S:L of 1:4 was used because a low substrate concentration leads to extremely diluted hydrolysates that are not feasible for industrial applications.

The mild conditions for the acid hydrolysis of mannan that were evaluated were not sufficient to efficiently break down the recalcitrance of this polysaccharide because 65–93% of the original mannose content remained in the solids recovered from the seed’s dilute-acid hydrolysis (Fig. 3). However, increasing the process’ severity by increasing the hydrolysis temperature, time or acid concentration could result in the formation of high quantity of degradation products from sugars and phenolic compounds, although there are different approaches for the removal of these products from the hydrolysate, including treatment using enzymes, vacuum evaporation of volatile compounds, chemical precipitation and adsorption on active charcoal of toxic compounds^61^. Nevertheless, in this study it was decided to avoid their formation during hydrolysis. Therefore, a sequential process of enzymatic hydrolysis of the recovered solids with the β-mannanases was evaluated to attempt to further release the mannose.

### Enzymatic hydrolysis for mannose release from recovered solids from acid hydrolysis

**Figure 4:**
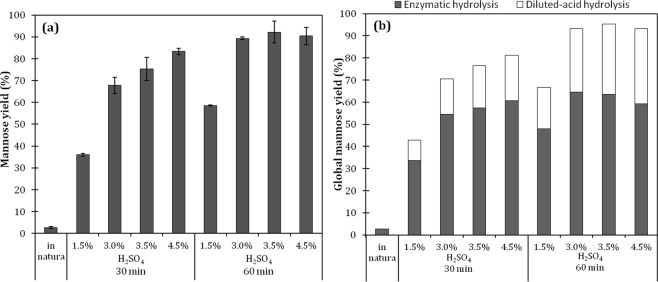
(**a**) Mannose yield obtained after 72 h of enzymatic hydrolysis of *in natura* and previously acid-hydrolyzed açaí seed milled samples; (**b**) Global mannose yield calculated in relation to the initial mannose content in the native seed. The assays were conducted with 2% solids and 400 IU of mannanase BGM “Amano” 10 per gram of sample.

After a preliminary screening of several commercial and lab-made enzyme preparations, the enzyme mannanase BGM “Amano” 10 (Amano Enzyme Inc., Nagoya, Japan) was selected as the most efficient for the hydrolysis of açaí seed’s mannan. This enzyme is indicated by the manufacture for applications related to coffee extraction and pulp bleaching, aiming at decreasing the viscosity of galactomannan, facilitating these processes. The enzyme preparation, which is commercially available as a powder, had activities of β-mannanase and β-mannosidase of 26,750 IU/g and 15.05 IU/g, respectively. Figure 4 shows the mannose yields obtained with the enzymatic hydrolysis of the acid-treated seed samples compared with the native milled seeds.

The native açaí seed sample was highly recalcitrant to the enzymatic attack, resulting in a less than 3% mannose yield. Nonetheless, after the material had been partially digested by sulfuric acid, it became much more susceptible to the attack of the β-mannanases, resulting in a 90% mannose yield for samples that were treated for 60 min with 3%, 3.5%, and 4.5% sulfuric acid. Consequently, the recovery of mannose could be substantially increased through a sequential process of dilute-acid hydrolysis followed by enzymatic hydrolysis, potentially reaching over 93% global mannose recovery for the most favorable conditions when using both the acid and enzymatic hydrolysis steps (Fig. 4b).

Data regarding the enzymatic hydrolysis of mannan into mannose is scarce in the literature because there are not many abundant agro-industrial residues rich in this polysaccharide. So far, there have been no studies in the literature exploring mannose production from açaí seeds, but there are some reports with other residues, such as PKC, copra meal, and spent coffee grounds. A study that evaluated the enzymatic hydrolysis of PKC, which contained 35.2% of mannan, reported that this residue was readily hydrolyzed into mannose with a mixture of two enzymes (Mannaway and Gammanase) with no previous treatment, resulting in 87% conversion of mannan into mannose after 96 h^45^. Most of mannan in PKC consists of a (1 → 4)-linked mannan with a low degree of substitution with galactose^62^, which is the same structure that we hypothesized for the mannan in açaí seeds. However, in the present study, native açaí seeds were poorly hydrolyzed by β-mannanases, reaching only 3% conversion of mannan to mannose, suggesting that these residues have distinctive mannan structures and/or the mannan is less accessible in açaí seeds because of interactions with other structural and nonstructural components.

**Figure 5:**
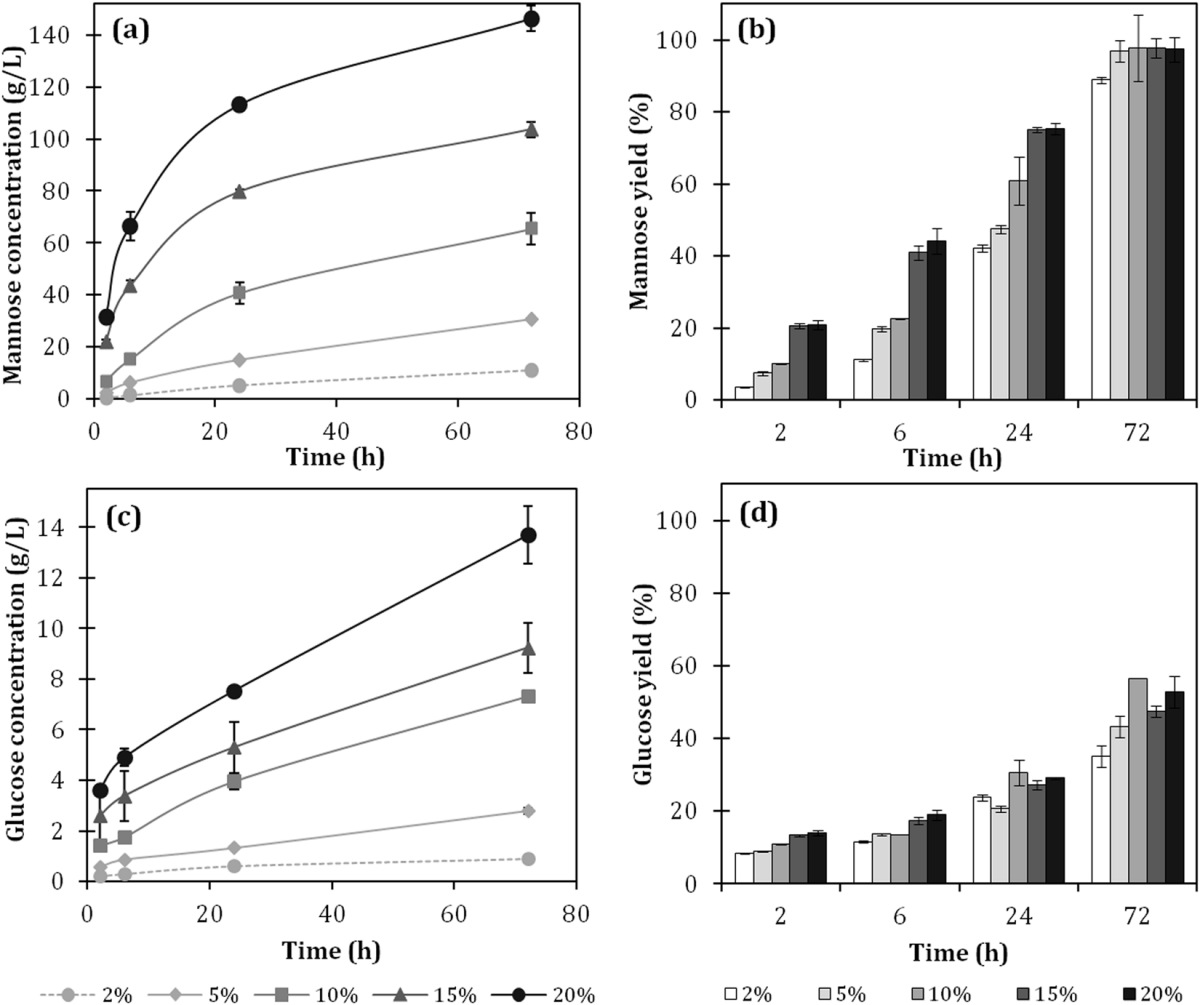
Enzymatic hydrolysis profile at the different solid contents of acid-hydrolyzed açaí seeds. (**a**) Mannose concentration; (**b**) mannose yield; (**c**) glucose concentration; (**d**) glucose yield. The assays were conducted with 400 IU of mannanase BGM “Amano” 10 per gram of sample. The samples were previously treated with 3% H_2_SO_4_ for 60 minutes at 121 °C.

The set of experiments presented in Fig. 4 were performed with a 2% açaí seed content (w/w), which led to high yield but also to hydrolysates containing low concentrations of mannose of about 11 g/L of at the best conditions. To have an effective industrial process, it is of the utmost importance to work on concentrated media to reduce the capital cost of equipment and the use of water. Therefore, the effect of solids loading on the enzymatic hydrolysis was evaluated in a range of 2–20% (Fig. 5). Samples treated with 3% acid for 60 min were selected for the assays because the seeds treated with 3%, 3.5%, and 4.5% of sulfuric acid were equally susceptible to β-mannanase attack (Fig. 4), and this condition has a lower impact on the use of H_2_SO_4_, formation of acidic effluents, and degradation products.

The enzymatic hydrolysis in the assays containing 2%, 5%, 10%, 15%, and 20% of acid-treated açaí seeds resulted in mannose concentrations, respectively, of 9.8, 30.6, 65.4, 103.7, and 146.3 g/L after 72 h. Regardless of the solid content evaluated (from 5–20%), the conversion of mannan content into mannose reached over 95% (Fig. 5b). The high yields achieved indicate that the β-endomannanase and β-mannosidase balance in the commercial enzymatic preparation used is adequate for the complete hydrolysis of açaí seed’s mannan. To the best of our knowledge, the mannose concentration reached in the assays with 20% solids is by far the highest reported in the literature for the enzymatic hydrolysis of an agricultural residue.

Only mannose and glucose were detected in the hydrolysates by HPLC analysis. At 72 h, the glucose concentrations reached 0.9, 2.8, 7.3, 9.2, and 13.7 g/L for the assays containing 2%, 5%, 10%, 15%, and 20% of acid-hydrolyzed açaí seed, respectively. It is very interesting to note that roughly a glucose:mannose ratio of 1:10 could be observed (See Suplementary Material). Considering that the enzyme used is a mannanase with nearly no cellulase activity, we hypothesize that the glucose released during enzymatic hydrolysis is derived from the mannan structure. The glucose:mannose ratio of 1:10 derived from mannan hydrolysis is in agreement with the definition of a “true” mannan, which relates to polysaccharides with more than 85–95% mannose content and a high degree of uniformity in the structure^37,47^.

The mannose and glucose concentrations obtained at 72 h of hydrolysis showed a linear correlation to the initial solid content, indicating that no significative inhibition effect took place during mannan hydrolysis. These data differ from what is typically reported for the enzymatic hydrolysis of cellulosic substrates because it has been shown that by increasing the substrate concentration, the corresponding yield decreases^63^. Although the 72 h mannose yields reached a plateau for all the solids content evaluated, the mannan conversion rate was faster in hydrolysis assays with higher solid contents, which also has an opposite effect to what is observed in the hydrolysis of fibrous cellulose-rich materials. This fact reinforces the observation that açaí seed mannan hydrolysis occurred in a pattern that differs greatly from the enzymatic hydrolysis of lignocellulosic materials by cellulases, which are affected by the “solids effect” including substrate effects, product inhibition, water content constraints, enzyme adsorption characteristics, and others^64^.

A similar evaluation was performed for the enzymatic hydrolysis of PKC using different substrate concentrations from 5–20% (w/v), reaching, at optimized conditions, a mannose concentration of 67.5 g/L. In agreement with our observation, Shukor *et al*.^65^ reported a direct increase in the production of simple sugars with an increase of the PKC content, which indicated that no substrate inhibition effect was taking place. However, the authors did not present a hydrolysis profile over time or the PKC characterization, which restricts other comparisons with the current study.

**Figure 6:**
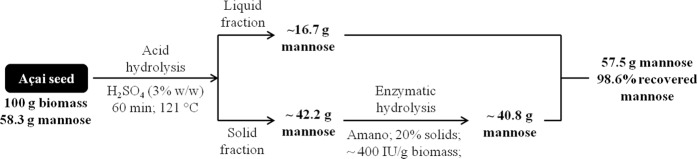
Mass balance of mannose production from milled açaí seeds treated with 3% H_2_SO_4_ for 60 min at 121 °C followed by enzymatic hydrolysis with BGM “Amano” 10 and 20% solid content.

A recent study has shown that 15 g of mannose could be obtained for every 100 g of spent coffee ground (SCG) after the removal of the non-saccharide content after delignification and defatting of SCG^43^. In the present study, 57.5 g of mannose could be obtained for every 100 g of *in natura* açaí seed, with a total mannose recovery of 98.6%. Figure 6 presents the mass balance of the overall process for the mannose release from açaí seeds considering the sequential process of dilute-acid hydrolysis and an enzymatic hydrolysis step with 20% solids. The results presented in the current study demonstrate that mannan from açaí seeds could be a low-cost source to produce mannose in high yields and concentrations. The development of this field could fill the present market demand for the cost-effective production of mannose and its derivatives^22^, which is hindered by the scarce sources of mannose and development of appropriate methods.

## Conclusions

In this pioneer study, a sequential process of dilute-acid and enzymatic hydrolysis of açaí seeds was developed to convert its high mannan content into mannose. Mannanases-catalyzed hydrolysis of acid-treated seeds resulted in 146 g/L of mannose and a 96.8% yield. To the best of our knowledge, this is by far the highest concentration of mannose reported for the enzymatic hydrolysis of an agricultural residue, which could open new perspectives for mannose use as a platform molecule. Finally, giving a proper destination to açaí seeds could add value to the whole açaí productive chain while promoting sustainable development in the Amazon region.

## Material and Methods

### Source of materials

Açaí seed samples were kindly donated by the company Açaí Amazonas Ltd. (Óbidos, Pará, Brazil). Samples were ground in a knife mill through a 2-mm screen (Universal cutting mill PULVERISETTE 19^®^, Fritsch, Idar-Oberstein, Germany). Mannanase BGM “Amano” 10 was kindly provided by Amano Enzyme Inc. (Nagoya, Japan). All other chemicals were purchased from commercial sources and used without any further purification.

### Characterization of açaí seeds: Determination of extractives, carbohydrates, and acid insoluble solids, X-ray diffraction, and seed fracture resistance analyses

For compositional analysis, *in natura* milled açaí seeds from lot 1 and 2 underwent an extraction process^66^ with some modifications. Approximately 2 g of the biomass were weighted into cellulose thimbles and extracted with water, which was followed by a 95% ethanol extraction; each extraction step was performed for at least 12 h. The procedure was carried out using six Soxhlet apparatus in parallel. By the end of the extraction, three of the thimbles were put in a 105 °C drying oven overnight to calculate the extractives by weight difference, while the other three were put in a 40 °C drying oven to be used in the following chemical characterization step. Then, 0.3 g of the dried, extractive-free *in natura* and acid-treated açaí seed samples were submitted to an acid hydrolysis process in two steps in triplicate, as described^67^. After this, the solutions were vacuum filtered through dried, preweighted Gooch crucibles. The acidic liquors were neutralized with CaCO_3_ and went through HPLC and HPAEC-PAD analyses, which are described below, for carbohydrate quantification. The crucibles containing the remaining solids were used for the determination of acid insoluble solids (AIS) and insoluble ash content, as described^67^.

X-ray diffraction (XRD) analyses of the milled açaí seed samples were performed using Bruker’s D8 Advance equipment (Bruker, Karlsruhe, Germany), Cu Kα radiation (“lambda” = 1.5418 angstroms), 40 kVA voltage and 40 mA current, a scanning angle in the range of 2 ≤ 2θ ≤ 60 degrees, and acquisition time of 0.6 s per step.

The evaluation of the seed fracture resistance was performed with an Instron® 3382 Floor Model Universal Testing System and the data acquisition was performed using the Instron Bluehill® Lite Software (Instron Company, Massachusetts, EUA). For the analyses, 20 core stones (seeds without the external fibrous layer) were pre-selected and classified into two groups according to their size and mass similarity. The equipment was coupled to a 10 KN load cell and the compression ratio was adjusted to 5 mm per min. The point which the compressive load curve had sudden drop in force was assumed as the minimal force necessary to promote the fracture in the seeds.

### Dilute-acid hydrolysis of açaí seeds for mannose release

Four sulfuric acid concentrations were evaluated for the dilute-acid hydrolysis step, corresponding to 1.5%, 3.0%, 3.5%, and 4.5% (% w/w). Each of these solutions was evaluated at a 30- and a 60-min residence time at 121 °C. Each condition was performed in at least four replicates in round-bottom hydrolysis tubes containing 4 g (dry weight) of the milled açaí seeds and 16 mL of the corresponding dilute-acid solution, resulting in a solid:liquid ratio of 1:4. The tubes were put in an autoclave for 30- or 60-min at 121 °C and then cooled in an ice bath. After this, 64 mL of water were added to the tubes, which were agitated for homogenization, and samples of the liquid streams were withdrawn, being then filtrated, neutralized, and prepared for chromatographic analysis, to determine the sugar and degradation products, as described below.

The solid contents of two of the four tubes were filtrated in preweighted fiber glass filters and put in an oven at 105 °C overnight to calculate the amount of mass transferred to the acidic liquid phase. The solid contents of the other two tubes were filtered and stored in the refrigerator until further use either for characterization of the chemical composition or for enzymatic hydrolysis assays. Prior to the characterization assays, the samples were dried at 40 °C until reaching less than 10% moisture.

The combined severity factor was calculated for each dilute-acid hydrolysis condition, which was evaluated based on the severity factor R_0_, which accounts for the effect of the temperature, residence time, and pH of the hydrolysates after the reaction, through the expression Log R_0_-pH, where Log R_0_ is given by Eq. (1)^55^:

1 $$\mathrm{Log}({{\rm{R}}}_{0})=\,\mathrm{Log}\lfloor {\rm{t}}\,{\rm{\times }}\,\exp (\frac{{\rm{T}}-100}{14.75})\rfloor $$

where *t* is the reaction time of the pretreatment in minutes, and *T* is the reaction temperature in °C.

### Enzyme activity measurements and enzymatic hydrolysis assays

The β-endomannanase activity of mannanase BGM “Amano” 10 was determined using a 0.5% locust bean gum (Sigma-Aldrich, St. Louis, MO, USA) solution as the substrate. The enzyme solution was diluted in a 50 mM sodium citrate buffer (pH 4.8), and an aliquot of 0.25 mL was mixed with 0.25 mL of the substrate solution and incubated in a water bath for 30 min at 50 °C. Then, 0.5 mL of 3,5-dinitrosalicylic acid (DNS) (Sigma-Aldrich, St. Louis, MO, USA), prepared according to Teixeira *et al*.^68^, was added to each tube after 30 min of incubation to stop the reaction, and the tubes were put in a boiling water bath for 5 min. The absorbance of the colored solutions was measured via spectrophotometer at a wavelength of 540 nm to quantify the reducing sugars. One unit of β-endomannanase was defined as the amount of enzyme required to release 1 µmol of reducing sugars equivalent to mannose in 1 min at 50 °C. β-mannosidase activity was determined by adding 100 μL of 10 mM of 4-nitrophenyl-β-D-mannopyranoside (Sigma-Aldrich, St. Louis, MO, USA) to 200 μL of a 0.5 M sodium citrate buffer (pH 4.8), 600 μL of distilled water, and 100 μL of an appropriately diluted enzyme sample. The assay was incubated at 50 °C for 10 min, and the reaction was stopped with the addition of 500 μL of 1.0 M sodium carbonate. The liberation of *p*-nitrophenol was monitored via spectrophotometer at a wavelength of 405 nm. One unit of β-mannosidase was defined as the amount of enzyme that released 1 μmol of *p*-nitrophenol for 1 min at 50 °C.

The enzymatic hydrolysis assays were performed in 50-mL flasks, with a total assay mass of 20 g containing 2–20% (w/w) of biomass (native seeds and seed samples after the acid hydrolysis) based on its dry weight, the enzyme solution in a 0.05 M sodium citrate buffer (pH 4.8), and 0.02% sodium azide. The amount of enzyme added was such that the β-mannanase activity load was 400 IU per gram of biomass, which was established in the preliminary assays. The flasks were incubated in a shaker at 50 °C and 200 rpm. Aliquots were withdrawn at 0, 2, 6, 24, 48, and 72 h and analyzed by HPLC for sugar quantification. Mannose yields were calculated according to Eq. (2).

2 $${\rm{Mannose}}\,{\rm{yield}}( \% )=\frac{({{\rm{C}}}_{{\rm{mannose}}}-{{\rm{C}}}_{{{\rm{mannose}}}_{0}})0.9\,}{(\frac{{{\rm{W}}}_{{\rm{seed}}}}{{{\rm{V}}}_{0}}){{\rm{F}}}_{{\rm{mannan}}}}\times 100$$

where C_mannose_ is the mannose concentration in the hydrolysates (g/L); ${{\rm{C}}}_{{{\rm{mannose}}}_{0}}$ is the initial mannose concentration in the hydrolysis assay; W_seed_ is the total weight of the seed in the hydrolysis assay (g); ${{\rm{V}}}_{0}$ is the initial volume of the liquid (L); F_mannan_ is the initial mass fraction of mannan in samples.

### Chromatographic conditions

Sugars and acetic acid were quantified by an Ultimate 3000 HPLC system (Thermo Scientific, Waltham, MA, USA) equipped with a refractive index detector RI-101 (SHODEX, Japan). For sugar quantification, an Aminex HPX-87P (300 × 7.8 mm, Bio-Rad Laboratories, Hercules, CA, USA) column was used, with a Carbo-P precolumn (Bio-Rad Laboratories, Hercules, CA, USA) and an inline deashing system (Bio-Rad Laboratories, Hercules, CA, USA). The mobile phase used ultrapure water at a flow rate of 0.6 mL/min with an oven temperature of 80 °C and detector temperature of 60 °C. The sugar composition of açaí seed samples and of acidic and enzymatic hydrolysates were also cross-checked by monosaccharides and disaccharides identification and quantification using a Thermo Scientific Dionex ICS-5000 system (Thermo Scientific, Waltham, MA, USA) using high-performance anion exchange chromatography with pulse amperometric detection (HPAEC-PAD). The guard cartridge and analytical column used were the CarboPac PA1 (4 mm × 50 mm, Thermo Scientific, Waltham, MA, USA) and CarboPac PA1 (4 mm × 250 mm, Thermo Scientific, Waltham, MA, USA). The column temperature was 15 °C, and the mobile phase was composed of phase A (type 1 reagent-grade deionized water) and phase B (300 mM NaOH solution). The gradient programs used for the separation were as follows: 0.0–32.0 min, 0% B; 32.0–32.1 min, 0–85% B; 32.1–42.0 min, 85% B; 42.0–42.1 min, 85–0% B; and 42.1–52.0 min, 0% B. The flow rate was 1.25 mL/min, and the injection volume was 5 µL. The system was also equipped with a postcolumn addition of 450 mM NaOH solution with a flow rate of 0.8 mL/min.

The acetic acid was quantified using an Aminex HPX-87H (300 × 7.8 mm, Bio-Rad Laboratories, Hercules, CA, USA) column with a Carbo-H precolumn (Bio-Rad Laboratories, Hercules, CA, USA) and an inline deashing system (Bio- Rad). The mobile phase was 5 mM H_2_SO_4_ at a flow rate of 0.6 mL/min with oven and detector temperatures of 30 °C and 45 °C, respectively. Furfural, hydroxymethylfurfural, and phenolic compounds (gallic acid, hydroxybenzoic acid, vanillin, ferulic acid, and cinnamic acid) were quantified with the diode array detector DAD-3000 (Thermo Scientific, USA). The column was a LiChroCART RP-18e (4.6 × 250 mm, Merck, Darmstadt, Germany) equipped with the precolumn LiChroCART RP-18e (4.0 × 4.0 mm, Merck, Darmstadt, Germany). The mobile phase was composed of phase A (type 1 reagent-grade deionized water) and phase B (methanol) at a flow rate of 0.4 mL/min, with oven temperatures of 30 °C and detector wavelengths of 280 and 320 nm. The gradient programs used for separation were as follows: 0.0–3.1 min, 15% B; 3.1–8.1 min, 65% B; 8.1–8.2 min, 95% B; 8.1–9.9 min, 95% B; 9.9–14.0 min, 0% B; 14.0–19.0 min, 15% B. Concentrations were quantified by external calibration.

## Supplementary information

Supplementary material

## Data Availability

All data generated or analyzed during this study are included in this article. Any additional information is available from the corresponding author on request.
