# Supplementary material for: High concentration and yield production of mannose from açaí (Euterpe oleracea Mart.) seeds via mannanase-catalyzed hydrolysis
Source: Sci Rep. 2019 Jul 29;9:10939. doi: 10.1038/s41598-019-47401-3 (PMC6662815; doi:10.1038/s41598-019-47401-3)
Supplement: Supplementary file 1 — Supplementary material [file 41598_2019_47401_MOESM1_ESM.docx]

**High concentration and yield production of mannose from açaí (*Euterpe oleracea* Mart.) seeds via mannanase-catalyzed hydrolysis**

Alvaro Ferreira Monteiro^1,+^, Ingrid Santos Miguez^1,2,+^, João Pedro R. Barros Silva^1^, and Ayla Sant’Ana da Silva^1,2,^*

^1^ Biocatalysis Laboratory, National Institute of Technology, Ministry of Science, Technology, Innovation and Communication, 20081-312, RJ, Brazil

^2^ Federal University of Rio de Janeiro, Department of Biochemistry, 21941-909, RJ, Brazil

^*^ ayla.santana@int.gov.br

^+^these authors contributed equally to this work

**Supplementary Material**

**Supplementary Table 1.** The compressive load (N) needed to rupture the açaí seeds of different size and mass.

|  | **Test specimens** | **Diameter** | **Mass** | | **Compressive load** | **Compressive extension** |
| --- | --- | --- | --- | --- | --- | --- |
|  |  | **(cm)** | **(g)** | **(N)** | | **(mm)** |
| **Group 1^*^** | TS-01 | 9.03 | 0.44 | 656 | | 1.09 |
|  | TS-02 | 9.07 | 0.45 | 914 | | 1.36 |
|  | TS-03 | 9.10 | 0.44 | 1155 | | 2.14 |
|  | TS-04 | 9.13 | 0.42 | 1021 | | 1.53 |
|  | TS-05 | 9.43 | 0.46 | 1243 | | 1.99 |
|  | TS-06 | 9.47 | 0.48 | 551 | | 1.43 |
|  | TS-07 | 9.73 | 0.52 | 775 | | 1.57 |
|  | TS-08 | 9.87 | 0.56 | 848 | | 1.38 |
|  | TS-09 | 9.93 | 0.55 | 1034 | | 1.71 |
|  | TS-10 | 10.23 | 0.57 | 1525 | | 2.56 |
| **Group 2^*^** | TS-11 | 11.22 | 0.86 | 851 | | 1.76 |
|  | TS-12 | 11.33 | 0.86 | 822 | | 0.98 |
|  | TS-13 | 11.37 | 0.86 | 1052 | | 1.72 |
|  | TS-14 | 11.50 | 0.84 | 937 | | 1.65 |
|  | TS-15 | 11.50 | 0.86 | 797 | | 1.28 |
|  | TS-16 | 11.57 | 0.87 | 1186 | | 2.00 |
|  | TS-17 | 11.60 | 0.92 | 1212 | | 1.93 |
|  | TS-18 | 11.80 | 0.97 | 961 | | 2.01 |
|  | TS-19 | 11.88 | 0.98 | 647 | | 1.75 |
|  | TS-20 | 12.10 | 0.97 | 1132 | | 2.02 |
|  |  |  |  |  | |  |

**^*^**Considering that the seeds are heterogeneous in size and mass, seed samples were pre-selected and classified into two groups according to their size and mass similarity. Group 1 represents the smaller seeds; while Group 2 contains the bigger ones.

**Supplementary Table 2.** Sugar concentration (g/L) released during the enzymatic hydrolysis with different solids loading, and the respective mannose/glucose ratio.

| Solids loading (%) | Sugar concentration (g/L) | | Man/Glu  Ratio |
| --- | --- | --- | --- |
|  | Mannose | Glucose |  |
| 20 | 146.3 | 13.7 | 10.7 |
| 15 | 103.7 | 9.2 | 11.3 |
| 10 | 65.4 | 7.3 | 9.0 |
| 5 | 30.6 | 2.8 | 10.9 |
| 2 | 10.9 | 0.9 | 12.1 |


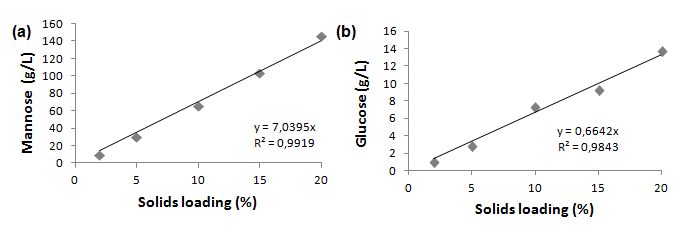


**Supplementary Figure 1.** (a) Mannose and (b) glucose concentration (g/L) released during the enzymatic hydrolysis versus solids loading in the assay.
